# Supplementary material for: Effect of Dry-Aging on Quality and Palatability Attributes and Flavor-Related Metabolites of Pork Loins
Source: Foods. 2021 Oct 19;10(10):2503. doi: 10.3390/foods10102503 (PMC8535753; doi:10.3390/foods10102503)
Supplement: Supplementary file 1 [file foods-10-02503-s001.zip › foods-1411870-supplementary.pdf]

Table S1. Demographic characteristics of consumers (n = 120) participated in the consumer sensory panels.

Demographic Questions	Response options	Frequency (%)
Gender	Female	63.3
	Male	35.8
	Other	0.9
Household Size	1 person	33.3
	2 people	23.3
	3 people	16.7
	4 people	13.3
	5 people	5.9
	>5 people	5.0
	Prefer not to disclose	2.5
Marital Status	Married	29.2
	Single	70.0
	Prefer not to disclose	0.8
Age	<20 years old	4.2
	20 to 29 years old	70.0
	30 to 39 years old	12.5
	40 to 49 years old	3.3
	50 to 59 years old	6.7
	>60 years old	3.3
Ethnic Origin	African-American	3.3
	Asian	30.0
	Caucasian/White	60.8
	Hispanic	2.5
	Mixed Race	2.5
	Other	0.9
Annual Household Income	<$25,000	35.8
	$25,000 to $34,999	7.5
	$35,000 to $49,999	4.2
	$50,000 to $74,999	7.5
	$75,000 to $99,999	10.0
	$100,000 to $149,999	10.8
	$150,000 to $199,999	5.0
	>$199,999	5.8
	Prefer not to disclose	13.4
When eating pork, what palatability trait is the most important to you?	Flavor	45.8
	Juiciness	26.7
	Tenderness	27.5
When eating pork, what degree of doneness do you prefer?	Medium-Rare	13.3
	Medium	31.7
	Medium-Well	25.8
	Well Done	26.7
	Not sure	2.5
How many times per week do you consume pork?	1 Time/week	28.33
	2 Times/Week	25.83
	3 Times/Week	15.83
	4 Times/Week	9.17
	5 Times/Week	5.83
	6-10 Times/Week	10.0
	>10 Times /Week	2.5
